# Feasibility of two levels of protein intake in patients with colorectal cancer: findings from the Protein Recommendation to Increase Muscle (PRIMe) randomized controlled pilot trial

**DOI:** 10.1016/j.esmoop.2024.103604

**Published:** 2024-06-26

**Authors:** K.L. Ford, M.B. Sawyer, S. Ghosh, C.F. Trottier, I.R. Disi, J. Easaw, K. Mulder, S. Koski, K.N. Porter Starr, C.W. Bales, J. Arends, M. Siervo, N. Deutz, C.M. Prado

**Affiliations:** 1 Department of Agricultural, Food & Nutritional Science, University of Alberta, Edmonton; 2 Department of Oncology, University of Alberta, Edmonton, Canada; 3 Department of Postgraduate Program of Anaesthesiology, Surgical Sciences and Perioperative Medicine, Faculdade de Medicina da Universidade de Sao Paulo, Sao Paulo, Brazil; 4 Durham VA Medical Centre, Durham; 5 Department of Medicine, Duke University, Durham, USA; 6 Department of Medicine I, Medical Center - University of Freiburg, Faculty of Medicine, University of Freiburg, Germany; 7 School of Population Health, Curtin University, Perth, Australia; 8 Center for Translational Research in Aging and Longevity, Texas A&M University, College Station, USA

**Keywords:** cancer, dietary intervention, low muscle mass, muscle loss, protein intake

## Abstract

**Background:**

Low muscle mass (MM) predicts unfavorable outcomes in cancer. Protein intake supports muscle health, but oncologic recommendations are not well characterized. The objectives of this study were to evaluate the feasibility of dietary change to attain 1.0 or 2.0 g/kg/day protein diets, and the preliminary potential to halt MM loss and functional decline in patients starting chemotherapy for stage II-IV colorectal cancer.

**Patients and methods:**

Patients were randomized to the diets and provided individualized counseling. Assessments at baseline, 6 weeks, and 12 weeks included weighed 3-day food records, appendicular lean soft tissue index (ALSTI) by dual-energy X-ray absorptiometry to estimate MM, and physical function by the Short Physical Performance Battery (SPPB) test.

**Results:**

Fifty patients (mean ± standard deviation: age, 57 ± 11 years; body mass index, 27.3 ± 5.6 kg/m^2^; and protein intake, 1.1 ± 0.4 g/kg/day) were included at baseline. At week 12, protein intake reached 1.6 g/kg/day in the 2.0 g/kg/day group and 1.2 g/kg/day in the 1.0 g/kg/day group (*P* = 0.012), resulting in a group difference of 0.4 g/kg/day rather than 1.0 g/kg/day. Over one-half (59%) of patients in the 2.0 g/kg/day group maintained or gained MM compared with 44% of patients in the 1.0 g/kg/day group (*P* = 0.523). Percent change in ALSTI did not differ between groups [2.0 g/kg/day group (mean ± standard deviation): 0.5% ± 4.6%; 1.0 g/kg/day group: −0.4% ± 6.1%; *P* = 0.619]. No differences in physical function were observed between groups. However, actual protein intake and SPPB were positively associated (β = 0.37; 95% confidence interval 0.08-0.67; *P* = 0.014).

**Conclusion:**

Individualized nutrition counselling positively impacted protein intake. However, 2.0 g/kg/day was not attainable using our approach in this population, and group contamination occurred. Increased protein intake suggested positive effects on MM and physical function, highlighting the potential for nutrition to attenuate MM loss in patients with cancer. Nonetheless, muscle anabolism to any degree is clinically significant and beneficial to patients. Larger trials should explore the statistical significance and clinical relevance of protein interventions.

## Highlights

• Patients were able to increase and sustain dietary protein intake from preintervention levels.
• Over one-half of patients in the 2.0 g/kg/day group maintained or gained MM with a 12-week targeted nutrition intervention.
• This work highlighted the potential for nutrition to attenuate MM loss in patients with cancer.

## Introduction

Cancer and anticancer treatment negatively affect muscle mass (MM),^1^ accelerating its loss,^2^ yet therapies to combat this prevalent condition remain to be elucidated.^3^ Low MM is associated with decreased physical function, reduced health-related quality of life, increased risk for treatment toxicity, delayed treatment, surgical complications, and shorter survival.^4^, ^5^, ^6^, ^7^, ^8^, ^9^, ^10^, ^11^, ^12^ Physical function (i.e. performance) and MM are commonly considered simultaneously in age- and disease-related muscle conditions^13^^,^^14^ and are endpoints of importance in oncology trials.^15^

Colorectal cancer (CRC) is the third most diagnosed cancer globally and the second cause of cancer-related mortality.^16^ Low MM is observed in ∼50% of patients with CRC,^17^ independent of body weight and weight loss.^10^ Muscle loss negatively affects prognosis and is the primary nutritional problem in patients with cancer.^3^ Without intervention, MM loss is observed in patients with early^18^ and advanced^19^^,^^20^ CRC. Prompt and continued optimization of nutritional status, including adapting to higher protein requirements,^21^ is crucial to prevent or minimize negative health outcomes such as MM loss and decreased physical function.^3^^,^^22^ In turn, nutritional interventions to treat muscle-related conditions or abnormalities remain the focus of several ongoing oncology trials.^23^

Protein intake is at the core of sustaining muscle health.^24^ Oncology nutrition guidelines recommend a protein intake of at least 1.0 g/kg/day and up to 1.5 g/kg/day if possible and include a call for research investigating the effects of increased protein (1.0-2.0 g/kg/day) on clinical outcomes.^21^ Despite skepticism, patients with cancer retain anabolic potential that is stimulated by protein intake.^25^ High-protein oral nutritional supplements or intravenous amino acids have been shown to increase the anabolic potential of muscle in patients with cancer, regardless of disease stage.^26^^,^^27^ The use of oral nutritional supplements with dietary advice reduced MM loss and prevalence of low MM in patients at nutritional risk following CRC resection.^28^ A systematic review found that MM maintenance during cancer treatment was possible with protein intake >1.4 g/kg/day, although patients who consumed <1.2 g/kg/day presented with muscle loss.^29^

Pragmatic approaches are needed to evaluate the feasibility of higher protein (2.0 g/kg/day) diets and their ability to promote MM maintenance during cancer therapy.^21^ Given the importance of protein intake, limited evidence on feasibility and impacts of 1.0 versus 2.0 g/kg/day, and in response to a call for research from oncology nutrition guidelines,^21^ we designed the Protein Recommendation to Increase Muscle (PRIMe) trial. The primary objective was to inform the feasibility of utilizing a diet containing 1.0 versus 2.0 g/kg/day of protein for the first 12 weeks of chemotherapy to halt MM loss. The secondary objective was to assess the effects of a 1.0 versus 2.0 g/kg/day diet on maintaining physical function.

## Patients and methods

### Trial design and ethical procedures

The PRIMe trial was a single-center, two-arm, open-label, randomized, controlled pilot trial conducted from August 2016 to April 2022. The protocol was published^30^ and the trial was registered as NCT02788955 on ClinicalTrials.gov.^31^ Clinical assessments of patients were completed at the Human Nutrition Research Unit,^32^ University of Alberta (Edmonton, AB, Canada). In response to the coronavirus disease 2019 (COVID-19) pandemic, recruitment was temporarily suspended from March to June 2020 as a result of public health restrictions. No patients were lost to follow-up or dropped out of the study during this period. Trial reporting was guided by the Consolidated Standards of Reporting Trials (CONSORT) extension for randomized pilot and feasibility trials.^33^ The study was approved by the Health Research Ethics Board of Alberta-Cancer Committee (HREBA.CC-15-0193) and complied with standards outlined in the Canadian Tri-Council Policy Statement: Ethical Conduct for Research Involving Humans.^34^ All patients provided written informed consent before study assessments.

### Study protocol

Ambulatory adults (18-85 years) with a recent diagnosis of stage II-IV CRC were recruited from the Cross Cancer Institute (Edmonton, AB, Canada). Patients were eligible if they could complete all baseline study assessments within 2 weeks of starting chemotherapy and had adequate hepatic and renal function. Reasons for exclusion were acute inflammation (assessed by neutrophil/lymphocyte ratio >5),^35^ ongoing (nontreatment related) nutrition impact symptoms (e.g. anorexia), severe dietary restrictions (e.g. veganism), a medical condition that impacted the ability to increase muscle (e.g. cachexia),^14^ active treatment for another cancer site, body weight >450 lb, pregnancy or lactation, a pacemaker *in situ*, insulin-dependent diabetes, or unstable thyroid disease.

**Figure 1:**
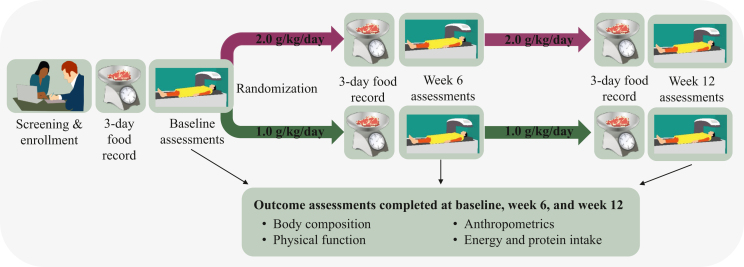
Graphical illustration of the Protein Recommendation to Increase Muscle (PRIMe) study protocol.

Patients were screened for eligibility and a study coordinator obtained approval from the treating medical oncologist before patients were approached with study information. Final eligibility verification was assessed through the electronic medical record of consented patients. Eligible patients completed a 3-day weighed food record to assess usual dietary intake. Patients were randomized to the 1.0 or 2.0 g/kg/day group in a 1 : 1 allocation ratio using blocks of six. An overview of the timeline and assessments is provided later and in Figure 1; detailed descriptions are presented in the trial protocol.^30^

### Nutrition intervention

A registered dietitian provided individualized nutrition counseling at baseline including dietary assessment and nutrition education. Patients received instructions to achieve a protein intake of 1.0 g/kg/day (minimum standard of care) or 2.0 g/kg/day.^21^ Prescribed diets were translated into an individualized daily meal pattern based on typical dietary intake.^36^ Meal patterns specified the number of food group ‘choices’ recommended per day to achieve individualized protein targets. An adapted version of the *Choose Your Foods: Food Lists for Weight Management* book developed by the Academy of Nutrition and Dietetics^37^ was provided to describe food group ‘choices’. For example, 1 protein ‘choice’ contained 7 g of protein and 1 milk group ‘choice’ contained 8 g of protein.^37^ Patients were provided a food scale and encouraged to weigh food portions. A multivitamin (natural product number: 80043120 or 80024313^38^; Supplementary Table S1, available at https://doi.org/10.1016/j.esmoop.2024.103604) was provided for daily consumption.

Patients were contacted by telephone weekly to assess adherence to protein intake recommendations using a 24-h dietary recall, chemotherapy-related nutrition impact symptoms, and self-reported body weight. The registered dietitian followed up with patients after 6 weeks and as needed. Patients were encouraged to meet their protein goal using a food-first approach but were provided whey protein powder [BENEPROTEIN; Nestlé Health Science, Toronto, ON, Canada) or PC Whey Protein Isolate (President’s Choice, Brampton, ON, Canada), Supplementary Table S2, available at https://doi.org/10.1016/j.esmoop.2024.103604] if weekly 24-h dietary recall suggested that protein intake recommendations were not being met, regardless of diet group allocation.

### Outcomes

Assessments were conducted at baseline, week 6, and week 12 using standardized procedures. MM by appendicular lean soft tissue index (ALSTI) was a surrogate marker of feasibility and the primary outcome; physical function by the Short Physical Performance Battery (SPPB) test was the secondary outcome.

### Patient characteristics and anthropometrics

Cancer stage was determined by the tumor–node–metastasis staging.^39^ A questionnaire was used to collect data on self-reported race and ethnicity, household income, and education level. Height was measured to the nearest 0.1 cm using a 235 Heightronic Digital Stadiometer (Quick Medical, Issaquah, WA) at baseline. Body weight was measured in triplicate to the nearest 0.1 kg using a calibrated digital scale (Health o meter Professional Remote Display; Sunbeam Products Inc., FL).

### Body composition

The feasibility of a 1.0 versus 2.0 g/kg/day diet to halt MM loss was assessed by a change in MM from baseline to week 12. Body composition was assessed by dual-energy X-ray absorptiometry [DXA; General Electric Lunar iDXA High Speed Digital Fan Beam Densitometer with Encore 13.60 software (General Electric Company, Madison, WI)]. Whole-body and regional estimates of lean soft tissue (LST), fat mass, and bone mineral content were derived. Fat-free mass was calculated by summing LST and bone mineral content. Appendicular LST (ALST) was divided by height (m^2^) to derive the ALSTI. Low MM was defined as ALSTI <7.0 kg/m^2^ for males and <5.5 kg/m^2^ for females.^13^

Computed tomography (CT) scans were obtained opportunistically from patients’ medical records to explore changes in skeletal muscle area at the tissue-organ level. The scan completed nearest to the date of baseline assessments was selected. The timeline of additional scans varied by patient, and thus the number of days between the CT scan and study assessment date was considered a covariate. Images from the third lumbar vertebra (L3) were selected based on correlation with whole-body skeletal muscle^40^ and landmarked using Slice-O-Matic 5.0 software (TomoVision, Magog, QC, Canada). Automated segmentation using Data Analysis Facilitation Suite software was conducted using −29 to 150 Hounsfield Units to identify skeletal muscle.^41^ The equation proposed by Shen and colleagues,^40^ *y* = 0.166*x* + 2.142, was used to estimate muscle volume from the cross-sectional area, considering the equation does not intersect the origin. Whole-body MM was estimated by converting from muscle volume using an assumed constant tissue density of 1.04 kg/L.

### Physical function

Physical function was assessed using the validated SPPB test (includes sit-to-stand, balance, and a timed 2.44-m walk).^42^ Each activity can score up to 4 points, for a total of 12 points.

### Nutritional intake

The feasibility of consuming a 2.0 g/kg/day protein diet during cancer treatment was assessed by weighed 3-day diet records. The nutrient composition was analyzed using The Food Processor Nutrition and Fitness Software (version 11.0.3 or version 11.7.217; ESHA Research, Salem, OR). Protein intake was compared with oncology nutrition recommendations (minimum: 1.0 g/kg/day; optimal: 1.2-1.5 g/kg/day)^21^ and the recommended dietary allowance (0.8 g/kg/day) for healthy populations.^43^

### Sample size

A formal sample size calculation was not required or carried out as this was a pilot study to assess the feasibility of the nutritional intervention.^33^ We enrolled 25 patients per diet group for a total sample size of 50.

### Statistical analysis

Statistical analyses were completed using IBM SPSS Statistics version 28 (International Business Machines Corporation, Armonk, NY) and GraphPad Prism version 9.3.1 for Windows (GraphPad Software, San Diego, CA). Variables were analyzed using the intention-to-treat (ITT) approach (i.e. based on diet group). Complete case analysis (i.e. only those who completed the study) and analyses based on the intervention received (i.e. amount of protein consumed) were also conducted.

No formal hypothesis testing was conducted as this was a pilot trial designed to assess the feasibility of a novel intervention and provide preliminary evidence that can be used to design a definitive trial.^33^^,^^44^ Data comparisons were carried out with an alpha level of 5% but caution was exercised when interpreting analyses as this study was not powered for drawing statistical inference but rather was intended to inform the feasibility of the intervention and a larger-scale trial. Thus 0.05 < *P* < 0.10 was noted as trending. Data are mean ± standard deviation or median and interquartile range (25th percentile-75th percentile) for non-normality unless otherwise indicated. Outliers were not removed. Normality was assessed by the Shapiro–Wilk test.

The ITT analysis explored change over time using generalized estimating equation models, a statistical technique used to account for between-subject (i.e. patient ID) and within-subject (i.e. time) correlation among responses seen in repeated measures studies.^45^^,^^46^ Generalized estimating equation models are valid under the assumption of data missing completely at random.^45^^,^^46^ An autoregressive model order 1 (AR-1) working correlation matrix was used to account for changes in correlations with time and an identity link function was used to form a linear relation between the dependent variable (ALSTI) and the predictor (i.e. diet group or actual protein intake). Models to assess MM loss (versus MM maintenance or gain) used a binomial distribution and logit link function. In addition, repeated measures analysis of covariance was used to assess outcomes while accounting for the baseline value of the outcome as a covariate. Mean adjusted differences, 95% confidence intervals (CIs), and eta-squared (η^2^; a measure of effect size) were presented. Comparisons between the 1.0 and 2.0 g/kg/day groups were also explored using independent samples *t*-test or Mann–Whitney *U* test (for non-normally distributed data) to assess mean differences at timepoints.

## Results

**Figure 2:**
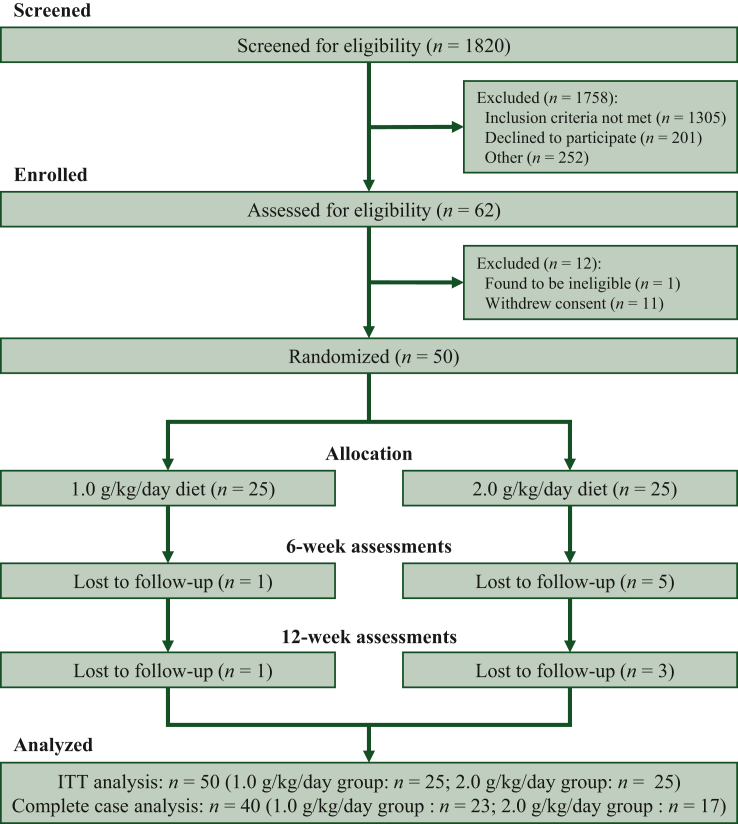
**Patient flow through the trial.** ITT, intention to treat.

Fifty patients completed baseline assessments, were randomized (*n* = 25 per group), and included in the ITT analysis (Figure 2). Primary and secondary outcomes are presented; no difference by ITT analysis was observed for exploratory measures (data not presented). Of the patients who did not complete the trial (*n* = 10), most (*n* = 6, 60%) were lost to follow-up before week 6. Loss to follow-up was more prevalent in the 2.0 g/kg/day group (*n* = 8 versus *n* = 2). Patient-reported reasons for discontinuing participation included feeling overwhelmed with managing a new cancer diagnosis or required time commitment.

**Figure 3:**
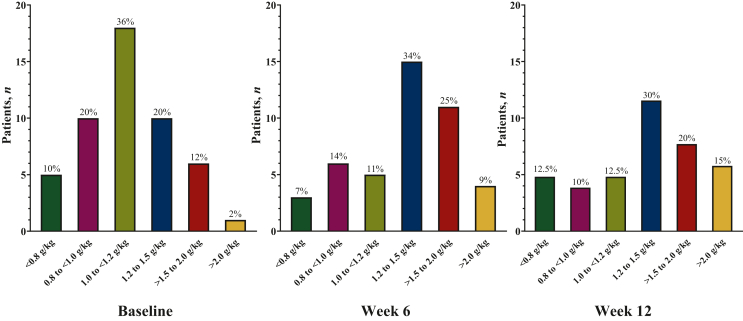
**Protein intake across study timepoints in patients with newly diagnosed colorectal cancer.** <0.8 g/kg: below RDA; 0.8 to <1.0 g/kg: meeting RDA; 1.0 to <1.2 g/kg: ESPEN minimum; 1.2-1.5 g/kg: ESPEN target; >1.5-2.0 g/kg: ESPEN high target; >2.0 g/kg: above ESPEN range. Baseline *n* = 50; week 6 *n* = 44; week 12 *n* = 40. ESPEN, European Society for Clinical Nutrition and Metabolism; g/kg, grams of protein per kilogram of body weight per day; RDA, recommended dietary allowance.

Patient characteristics are described in Table 1. Before the dietary intervention, most (*n* = 33, 66%) recorded protein intake below the oncology nutrition guidelines target (i.e. <1.2 g/kg/day) while one patient (2%) had protein intake >2.0 g/kg/day (Figure 3). Considering patients who completed the trial, 32.5% (13/40) were consuming an optimal amount of protein (≥1.2 g/kg/day)^21^ before the trial intervention compared with 65% (26/40) after 12 weeks of dietary intervention (*P* = 0.002). Protein powder was supplied to 29.5% of patients (*n* = 13; 2.0 g/kg/day group: *n* = 10 and 1.0 g/kg/day group: *n* = 3) at week 6 and 35% (*n* = 14; 2.0 g/kg/day group: *n* = 11 and 1.0 g/kg/day group: *n* = 3) at week 12 to obtain the anticipated protein intake per assigned group.

**Table 1:** Baseline characteristics of 50 patients with colorectal cancer

Characteristics	Total (*N* = 50)	1 g/kg/day (*n* = 25)	2 g/kg/day (*n* = 25)	*P* value
**Demographic and clinical**				
Age (years)	57 ± 11	57 ± 13	58 ± 9	0.840
Sex, *n* (%)				0.564
Female	20 (40)	9 (36)	11 (44)	
Male	30 (60)	16 (64)	14 (56)	
Disease stage, *n* (%)				0.747
II/III	37 (74)	19 (76)	18 (72)	
IV	13 (26)	6 (24)	7 (28)	
Chemotherapy, *n* (%)^a^				0.158
Capecitabine alone	6 (12)	5 (20)	1 (4)	
Oxaliplatin based	34 (68)	14 (56)	20 (80)	
Irinotecan based	10 (20)	6 (24)	4 (16)	
Ostomy, *n* (%)				0.208
Yes	14 (28)	9 (36)	5 (20)	
No	36 (72)	16 (64)	20 (80)	
**Anthropometrics**				
Weight (kg)	80.0 ± 18.0	77.3 ± 15.5	82.8 ± 20.1	0.284
Body mass index (kg/m^2^)^c^	27.0 (22.9-31.3)	23.9 (22.1-30.8)	28.6 (24.5-32.8)	0.143
**Body composition**				
Fat mass (kg)				
Total	28.1 ± 10.6	26.6 ± 10.3	29.5 ± 10.8	0.337
Female	30.3 ± 10.4	30.6 ± 11.5	30.0 ± 9.9	0.902
Male	26.6 ± 10.6	24.4 ± 9.2	29.1 ± 11.9	0.226
Fat mass (%)				
Total	34.5 ± 9.0	33.9 ± 9.1	35.1 ± 9.1	0.619
Female^c^	43.3 (35.5, 46.2)	41.0 (36.4, 46.8)	43.5 (31.2, 45.8)	0.941
Male	30.2 ± 7.2	29.5 ± 7.0	31.0 ± 7.6	0.557
Fat-free mass (kg)				
Total^c^	49.4 (43.9-61.0)	51.2 (44.1-57.7)	48.5 (43.3-63.9)	0.793
Female	41.8 ± 5.1	40.9 ± 5.8	42.6 ± 4.6	0.481
Male^d^	58.7 ± 10.2	56.1 ± 7.1	61.7 ± 12.4	0.151
Fat-free mass (%)				
Total	66.5 ± 9.0	66.1 ± 9.1	64.9 ± 9.1	0.619
Female^c^	56.7 (53.8-64.5)	59.0 (53.2-63.6)	56.5 (54.2-68.8)	0.941
Male	69.8 ± 7.2	70.5 ± 7.0	69.0 ± 7.6	0.557
ALST (kg)				
Total^c^	20.6 (17.7-26.1)	22.1 (17.8-24.0)	20.3 (17.2-27.3)	0.869
Female	17.1 ± 2.5	17.0 ± 2.7	17.3 ± 2.4	0.767
Male	25.3 ± 5.4	24.0 ± 3.9	26.7 ± 6.5	0.168
ALSTI (kg/m^2^)				
Total	7.4 ± 1.5	7.3 ± 1.2	7.7 ± 1.8	0.262
Female	6.5 ± 1.0	6.5 ± 1.0	6.6 ± 1.0	0.819
Male	8.1 ± 1.5	7.7 ± 1.1	8.6 ± 1.8	0.079
Low muscle mass, *n* (%)^e^				0.480
Yes	10 (20)	6 (24)	4 (16)	
No	40 (80)	19 (76)	21 (84)	
**Physical function**				
Short Physical Performance Battery test score^b^^,^^c^, 0-12	11.0 (10.0-12.0)	11.0 (10.5-12.0)	11.0 (9.5-12.0)	0.632
**Energy and protein intakes**				
Energy intake (kcal)^d^	2147 ± 635	2116 ± 755	2178 ± 500	0.736
Energy intake (kcal/kg)^c^	26 (22-33)	26 (22-35)	26 (21-33)	0.648
Protein intake (g)	88.7 ± 28.5	85.0 ± 33.2	92.4 ± 22.9	0.365
Protein intake (g/kg)^c^	1.0 (0.9, 1.4)	1.0 (0.9, 1.3)	1.1 (1.0, 1.4)	0.577

Data are presented as mean ± standard deviation if normally distributed or as median (25th percentile-75th percentile) in cases of non-normality. Independent samples *t*-test were used to assess the mean difference between groups for continuous variables and the chi-square test was used for the difference between categorical variables.

ALST, appendicular lean soft tissue; ALSTI, Appendicular Lean Soft Tissue Index.

a Fisher’s exact test applied (chi-square assumption violated).

b Lower scores indicate decreased physical function.

c Mann–Whitney *U* test due to non-normal distribution of one or more groups.

d Welch *t*-test was used due to the heterogeneity of variances.

e Low muscle mass was defined as ALSTI <7.0 kg/m^2^ for males and <5.5 kg/m^2^ for females.

### Protein and energy intakes

**Figure 4:**
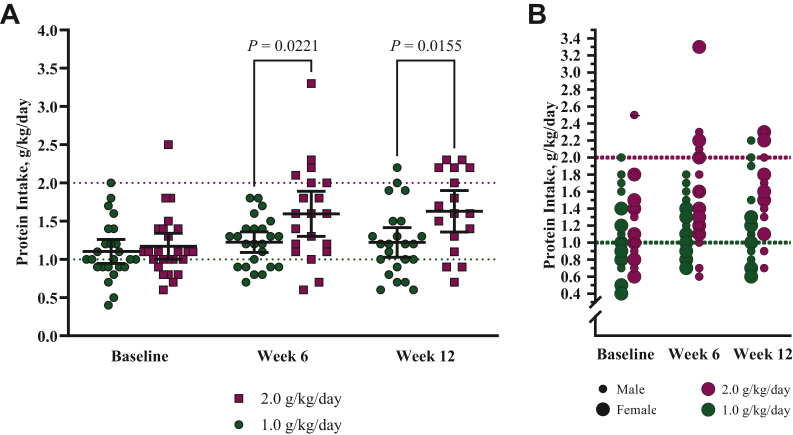
**Protein intake by (A) study arm and timepoint and (B) study arm, timepoint, and sex.** Data are presented as mean. Green circles represent the 1.0 g/kg/day protein diet group; purple squares represent the 2.0 g/kg/day protein diet group. The dotted lines represent the target protein intake per study group. Smaller circles represent males; larger circles represent females. Data point with a through line indicates an extreme outlier (significance was not impacted by the extreme outlier). Differences between study arms at each timepoint were assessed by independent samples *t*-test or Mann–Whitney *U* test for non-normality. Difference between baseline and week 12 intake by the study arm was assessed by paired samples *t*-test; 1.0 g/kg/day: 0.1 ± 0.4 g/kg/day (*P* = 0.357) and 2.0 g/kg/day: 0.5 ± 0.4 g/kg/day (*P* < 0.001). g/kg/day, grams of protein per kilogram of body weight per day. Baseline: *n* = 50 (1.0 g/kg/day group: *n* = 25; 2.0 g/kg/day group: *n* = 25); 6 weeks: *n* = 44 (1.0 g/kg/day group: *n* = 24; 2.0 g/kg/day group: *n* = 20); 12 weeks: *n* = 40 (1.0 g/kg/day group: *n* = 23; 2.0 g/kg/day group: *n* = 17).

Being in the 2.0 g/kg/day group resulted in 0.3 g/kg/day greater protein intake compared with the 1.0 g/kg/day group (β = 0.3; 95% CI 0.0-0.5; *P* = 0.019) across timepoints. The protein intake goal in the 2.0 g/kg/day group was achieved by 30% of patients (*n* = 6) at week 6 and by 35.3% (*n* = 6) at week 12 while 8.7% of patients (*n* = 2) in the 1.0 g/kg/day group also consumed ≥2.0 g/kg/day of protein at week 12. Change in protein intake differed between groups (1.0 g/kg/day: 0.1 ± 0.4 g/kg/day; 2/0 g/kg/day: 0.6 ± 0.4 g/kg/day; *P* < 0.001) at week 12. Protein intake in the 2.0 g/kg/day group was 1.6 ± 0.5 g/kg/day compared with 1.2 ± 0.4 g/kg/day in the 1.0 g/kg/day group (*P* = 0.012) at week 12 (Figure 4A). Thus protein intake differed between groups by 0.4 g/kg/day (versus intended 1.0 g/kg/day) at week 12. Protein intake by diet group and sex is illustrated in Figure 4B.

Percent change in protein intake since baseline trended toward a difference at week 6 (Supplementary Figure S1A, available at https://doi.org/10.1016/j.esmoop.2024.103604). At week 12, percent change in protein intake differed between groups (2.0 g/kg/day: 54.5% ± 48.6%; 1.0 g/kg/day: 10.2% ± 33.7%; *P* = 0.002) and percent change in energy intake trended toward being greater in the 2.0 g/kg/day group (Supplementary Figure S1B, available at https://doi.org/10.1016/j.esmoop.2024.103604). After adjusting for baseline protein intake as a covariate, the 2.0 g/kg/day group (1.7 g/kg/day; 95% CI 1.5-1.9 g/kg/day) had higher protein intake than the 1.0 g/kg/day group (1.2 g/kg/day; 95% CI 1.0-1.4 g/kg/day; *P* < 0.001) at week 12. Baseline protein intake accounted for 26% of variability in protein intake at week 12 (*P* < 0.001; η^2^ = 0.26).

### Body composition and anthropometrics

**Figure 5:**
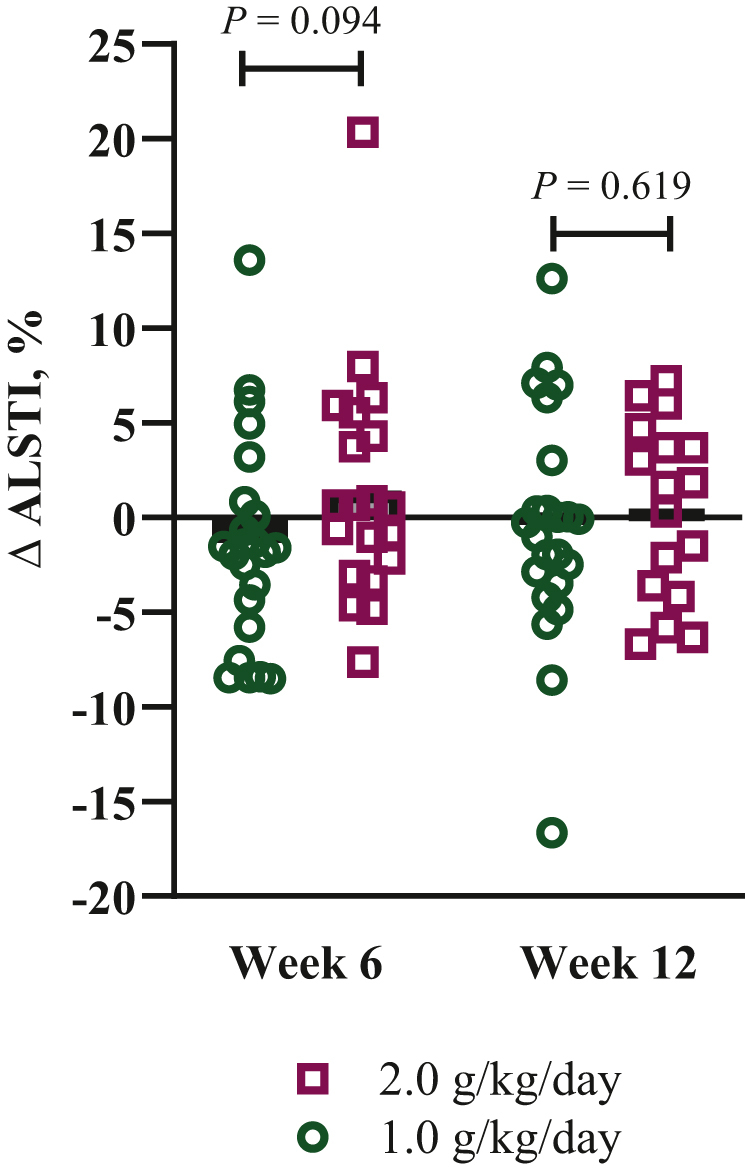
**Percent change since baseline in muscle mass estimated by ALSTI.** Each data point represents a patient. Black bars represent the group mean. Differences between study groups were assessed by independent samples *t*-test or Mann–Whitney *U* test in the case of non-normality. ALSTI, Appendicular Lean Soft Tissue Index; 6 weeks: *n* = 44 (1.0 g/kg/day group: *n* = 24; 2.0 g/kg/day group: *n* = 20); 12 weeks: *n* = 40 (1.0 g/kg/day group: *n* = 23; 2.0 g/kg/day group: *n* = 17).

Half of all patients (*n* = 20) maintained or gained MM at week 12. Considering sex, odds of MM loss were 86.3% lower for females in the 2.0 g/kg/day group compared with females in the 1.0 g/kg/day group (odds ratio 0.14; 95% CI 0.02-0.90; *P* = 0.038). Odds of MM loss (versus maintained or gained) did not differ between diet groups (data not shown). At week 12, the difference in MM between groups trended toward significance (ALSTI 2 g/kg/day: 8.2 ± 1.8 kg/m^2^; 1 g/kg/day: 7.2 ± 1.2 kg/m^2^; *P* = 0.065). Percent change in ALSTI from baseline did not differ between groups (Figure 5). At week 12, females in the 2.0 g/kg/day group had a greater percent change in ALSTI than females in the 1.0 g/kg/day group (Supplementary Figure S2, available at https://doi.org/10.1016/j.esmoop.2024.103604). Baseline MM accounted for 94% of the variance in follow-up measures of MM (*P* < 0.001; η^2^ = 0.94).

**Figure 6:**
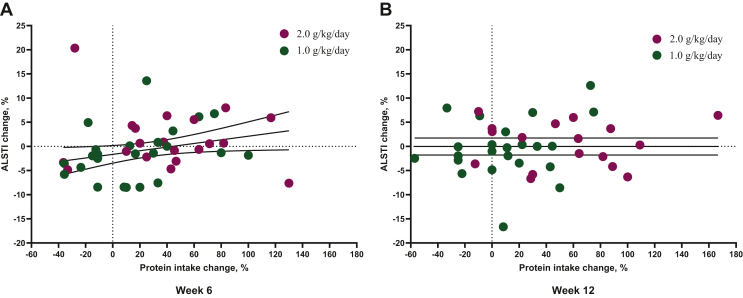
**(A, B) Percent change since baseline in ALSTI plotted against percent change in protein intake since baseline at (A) week 6 and (B) week 12 by study arm.** Each data point represents a participant. The hashed lines are a marker of no change. ALSTI, Appendicular Lean Soft Tissue Index.

Irrespective of diet group allocation, percent change in MM since baseline trended toward a positive association with actual protein intake and suggested that an increase of 1.0 g/kg/day protein may result in a 1.6% increase in ALSTI (β = 1.57; 95% CI −0.24 to 3.39; *P* = 0.090). The percent change in MM as a function of the percent change in protein intake is shown in Figure 6. Changes in weight, fat mass, fat-free mass, and ALST as a percent of baseline are shown in Supplementary Figure S3A, B.

Skeletal muscle area (cm^2^) was not different between diet groups when adjusting for baseline muscle area and time between study visit and CT scan. When accounting for sex and time between baseline visit and CT scan, the 2.0 g/kg/day group had 15.3-cm^2^ greater skeletal muscle area compared with the 1.0 g/kg/day group (*P* = 0.030), which equals ∼2.6-kg estimated whole-body MM. The median number of days between the CT scan and baseline assessment was 69 (25th percentile: –78 days; 75th percentile: 283 days).

### Physical function

An increase in 1.0 g/kg/day protein resulted in an increase in the SPPB test score of 0.4 points (β = 0.4; 95% CI 0.1-0.7; *P* = 0.014). For the sit-to-stand component of the SPPB test, a measure of lower extremity strength, scores increased by 0.4 points for every 1.0 g/kg/day increase in protein intake (β = 0.4; 95% CI 0.1-0.6; *P* = 0.006). The ITT analysis found no difference between diet groups for the SPPB total test score [25th percentile: 2.0 g/kg/day: 11 (11, 12); 75th percentile: 1.0 g/kg/day: 12 (11, 12); *P* = 0.745] or subcomponent scores at week 12 (Supplementary Figure S4, available at https://doi.org/10.1016/j.esmoop.2024.103604).

## Discussion

We show in this first randomized controlled pilot trial the feasibility and potential impact of different (higher) levels of protein intake on MM and physical function in patients receiving chemotherapy for CRC. Reaching a 2.0 g/kg/day diet of protein for 12 weeks supported by individualized nutritional counseling from a registered dietitian was not achievable at the group level, although was attained by 20% of patients who completed the trial. When protein intake was considered, regardless of diet group allocation, increased consumption showed potential for positive effects on MM maintenance, anabolism, and physical function. After 12 weeks of targeted nutrition intervention, one-half of the patients maintained or gained MM. This avoidance of muscle loss is notable given the catabolic effects of cancer and anticancer treatment.^47^ Using opportunistic CT scans, greater skeletal muscle area was observed in patients in the 2.0 g/kg/day group when accounting for sex and time. Overall, individualized nutrition counselling positively impacted protein intake, and it was feasible for patients to increase and sustain dietary protein intake from preintervention levels, although attaining 2.0 g/kg/day was challenging.

### Can we feasibly increase protein intake in patients with cancer?

Our study used pragmatic components to gain insights into the feasibility of implementing a targeted nutrition intervention for 12 weeks in an outpatient oncology setting. Patients over- and underconsumed protein in comparison to their assigned protein intake goal (1.0 versus 2.0 g/kg/day), as suggested by a mean difference of 0.4 g/kg/day intake between groups. The observed difference in protein intake between groups is likely insufficient to surmount a physiological response over 12 weeks given the catabolic, anorectic, and fatigue effects of chemotherapy. Nonetheless, our findings highlight the promising impact of targeted nutrition intervention and nutrition counseling on protein intake and MM in patients with cancer. These findings add to the literature suggesting that increased protein intake has the potential to positively affect MM.^29^

Nutritional support (e.g. information/resources on managing nutrition-impact symptoms, high-protein recipes, protein powder supplements, and/or select availability of precooked frozen meat products) provided to patients extended beyond the standard of care.^48^ Protein intake in patients with cancer varies; many patients do not meet the minimum recommended intake of 1.0 g/kg/day or the target of 1.2 g/kg/day^49^, ^50^, ^51^, ^52^ as further confirmed by our cohort at baseline. Notwithstanding the mean intake in our cohort (1.1 g/kg/day), individual protein consumption was highly variable and 30% of patients did not attain the minimum recommended intake before intervention. These findings are similar to those previously reported in the literature, although most studies have been conducted in patients with advanced cancers.^50^, ^51^, ^52^

The threshold of protein intake to maintain MM in cancers associated with muscle loss is likely 1.4 g/kg/day,^29^ implying that current protein recommendations in cancer^21^ may not be sufficient to support muscle health considering food intake alone. After 12 weeks of targeted nutrition intervention, the mean protein intake was 1.4 g/kg/day, and baseline protein intake accounted for ∼25% of variation in observed protein intake at week 12. Over the course of the intervention, protein intake in the 2.0 g/kg/day group increased by 0.6 g/kg/day in patients who completed the trial. For a person weighing 80 kg, that represented an increase in protein intake of 48 g/day (e.g. ∼6 oz or 171 g of meat), and 40% of a recommended target level for people with cancer (e.g. 1.5 g/kg/day). In response to a call for research investigating 2.0 g/kg/day protein intake,^21^ we showed that this level of intake was not feasible in patients being treated for CRC, even when they received individualized dietitian counseling and provision of whey protein (the latter as requested). This finding is notable considering that our cohort had adequate functional status at baseline based on the inclusion criteria of the trial.

Our choice of the 1.0 g/kg/day group potentially impacted findings as we felt ethically compelled to use the minimal recommended protein intake as the target for the active control group. This approach likely improved patient outcomes in the 1.0 g/kg/day group but lessened our ability to detect differences between groups, especially given that patients in the 1.0 g/kg/day group increased their intake. There was only a 0.4 g/kg/day difference in protein intake between groups (versus the intended 1.0 g/kg/day difference) and there was both over- and under-consumption of protein relative to protein intake goals.

### Can we increase muscle mass in patients with cancer?

After 12 weeks of targeted nutrition intervention, MM assessed by ALSTI trended toward being different between groups at week 12, which could be explained by the exploratory nature of the study (i.e. without an *a priori* power calculation). Nonetheless, the probable increase in MM with every 1.0 g/kg/day increase in protein intake is clinically relevant^3^^,^^14^^,^^21^ and achievable (maximum change to protein intake observed at week 12 was 1.2 g/kg/day). This finding suggests that muscle anabolism in cancer is possible and was stimulated by diet alone in our study. To put these findings into context, a person weighing 80 kg with an ALSTI of 7.26 kg/m^2^ could potentially increase their ALSTI to 7.42 kg/m^2^ by consuming an additional 80 g of protein per day (e.g. ∼10 oz or 283 g of meat). Although this increase in ALSTI may seem negligible, it represents muscle anabolism in patients with cancer,^3^^,^^21^^,^^53^ which is clinically relevant and could potentially reverse a diagnosis of low MM. This is in contrast to findings from studies with a similar timeline alignment of MM loss ranging from 0.1% over 12 weeks in patients with early stage disease^18^ to 5.6% over 12 weeks in patients with advanced CRC.^19^^,^^20^ Preservation of MM is essential to avoid detrimental and rapid loss of muscle that requires significantly more time to rebuild,^20^^,^^54^^,^^55^ a concept that has been described using the analogy of a wildfire.^22^

Previous research on the anabolic potential of skeletal muscle during cancer has been controversial; studies have indicated both anabolic resistance^27^^,^^56^ and retained anabolic potential.^25^^,^^27^^,^^57^^,^^58^ Our findings are particularly important given that nutritional status and interventions are not often a point of focus at the time of a cancer diagnosis and that skepticism surrounding the importance of nutrition is prevalent in the clinical setting.^3^ Notably, MM maintenance alone (without anabolism) is considered a positive health outcome in oncology.^3^^,^^21^^,^^53^ However, body weight change is commonly used as a marker of nutritional status in the clinical setting, given limited access to DXA or other body composition assessment techniques.^10^ Body weight changes in our study showed that patients in the 2.0 g/kg/day group trended toward having a higher percent change in weight over the first 6 weeks. These findings suggest that this intervention may be useful for weight-losing patients and also highlight the importance of considering interventions with a physical activity component (which notably can increase protein requirements).

Secondary analyses of baseline data (published elsewhere) showed that DXA-derived estimates of body composition had the strongest agreement with the criterion multicompartment model.^59^ Our assessment of MM used DXA-derived measures (i.e. indirect measures). ALST accounts for >75% of whole-body muscle^60^ and provides a surrogate marker to whole-body MM.^61^ At the whole-body level, LST from DXA includes organ, fibrotic, and other tissues.^62^ Thus if abnormal tissue (e.g. tumor) is present within the trunk of the body, it is especially important to consider ALST as a surrogate marker of whole-body MM. For direct assessment of MM, opportunistically acquired CT scans found that, when accounting for sex and length of time from study assessments to CT scan, patients in the 2.0 g/kg/day group had more skeletal muscle at L3 which equated to ∼2.6 kg MM at the whole-body level.^40^ Findings were not significant when based on actual protein intake.

### Can we increase physical function in patients with cancer?

Physical function was assessed by the SPPB test which also provides an indicator of low MM severity.^13^ The established cut-off point for low performance by the SPPB test in a healthy population is ≤8 points,^13^ which questions the clinical relevance of the change in score (0.4 points) that was observed with increased protein intake in our cohort. The SPPB is designed for patients >70 years^63^ although it has been compared between younger and older patients with cancer and no differences in test scores were observed.^64^ Patients in our cohort had adequate physical function (baseline SPPB score: mean ± standard deviation 10.7 ± 1.5) that appeared to be positively impacted by increased protein intake. Improvement in sit-to-stand, a measure of lower extremity strength, was noted despite 36% (*n* = 18) of the cohort scoring 12 (maximal score) on the SPPB at baseline, limiting their ability to show improvement.

### Strengths and limitations of the trial

Our choice to include noncachectic patients with metastatic disease who were receiving any type of chemotherapy likely captures the broad spectrum of CRC outpatients. The semipragmatic approach to the dietary intervention provided insight into the feasibility of implementing dietary change in this population and aligns with strategies to optimize clinical nutrition research.^65^ This strength is coupled with the limitation that a lack of a placebo-controlled and/or blinded approach may have induced contamination in our 1.0 g/kg/day group. Future trials could consider the incorporation of a more standardized approach to increased protein intake (e.g. using medical nutrition therapy). Notably, the median protein intake before the intervention was 1.0 g/kg/day which may have masked potential effects. Another key consideration that speaks to the feasibility of the intervention is the fourfold higher dropout observed in the 2.0 g/kg/day group. Reaching statistical significance was never the intention of the trial, given its exploratory nature, and does not signify a negative trial in this case. We sought to understand the feasibility and impact of diet alone, as increased physical activity would increase nutritional needs. We acknowledge that this approach may be viewed as a limitation because exercise is known to positively impact MM in cancer and may have increased anabolic response.^66^ Lastly, data collection spanned >5 years which highlights the challenges in recruiting patients for a dietary intervention trial near the time of diagnosis and during a global pandemic.

### Future directions and conclusion

Findings from this work suggest potential benefits of the intervention related to protein intake and muscle, which are clinically relevant considerations in patients with cancer. This feasibility pilot trial can be used to design larger independent and definitive trials to assess the impact of protein intake on MM and physical function in patients with cancer. The intervention should be adapted to the patient population, and account for relevant factors such as nutrition impact symptoms and ability to implement dietary changes. Exploratory outcome measures, including nutrition impact symptoms, will be detailed in secondary analyses to provide further insight. Loss of MM increases with age,^67^ regardless of disease presence. Age at the time of diagnosis in patients with CRC is trending younger on a global scale,^16^ especially in North America^68^; thus the impact of the disease on MM in younger adults with CRC is an area that warrants further investigation. Besides, the prevalence of advanced disease stage at the time of diagnosis is postulated to increase secondary to delayed screening and treatment caused by the COVID-19 pandemic^69^ and should continue to be a consideration. Lastly, the incorporation of patient-reported outcomes is essential to better understanding the patient’s perspective and the impact of the intervention on their health condition and was explored through patient interviews in a secondary qualitative analysis.^70^

Overall, our findings highlight the potential for nutritional intervention alone to halt muscle loss during cancer. Muscle anabolism to any degree is clinically significant and may be the difference between low and normal MM. Definitive and adequately powered well-controlled randomized trials that include robust assessment techniques are needed to confirm our findings and to determine the optimal protein dose, in terms of both feasibility and efficacy, to recommend muscle support in patients undergoing anticancer treatment.
