# Supplementary material for: Feasibility of two levels of protein intake in patients with colorectal cancer: findings from the Protein Recommendation to Increase Muscle (PRIMe) randomized controlled pilot trial
Source: ESMO Open. 2024 Jun 26;9(7):103604. doi: 10.1016/j.esmoop.2024.103604 (PMC11260369; doi:10.1016/j.esmoop.2024.103604)
Supplement: Supplementary Tables 1 and 2 and Figures 1-4 [file mmc1.pdf]

Supplementary Materials

Feasibility and Impact of Two Levels of Protein Intake in Patients with Colorectal Cancer: Findings from the Protein Recommendation to Increase Muscle (PRIMe) trial

K. L. Ford[1], M. B. Sawyer[2], S. Ghosh[2], C. F. Trottier[1], I. R. Disi[1,3], J. Easaw[2], K. Mulder[2], S. Koski[2], K. N. Porter Starr[4,5], C. W. Bales[4,5], J. Arends[6], M. Siervo[7], N. Deutz[8], C. M. Prado[1*]

[1]Department of Agricultural, Food & Nutritional Science, University of Alberta, Edmonton, Canada

[2]Department of Oncology, University of Alberta, Edmonton, Canada

[3]Department of Postgraduate Program of Anaesthesiology, Surgical Sciences and Perioperative Medicine, Faculdade de Medicina da Universidade de Sao Paulo, Sao Paulo, Brazil

[4]Durham VA Medical Centre, Durham, United States

[5]Department of Medicine, Duke University, Durham, United States

[6]Department of Medicine I, Medical Center - University of Freiburg, Faculty of Medicine, University of Freiburg, Germany

[7]School of Population Health, Curtin University, Perth, WA, Australia

[8]Center for Translational Research in Aging and Longevity, Texas A&M University, College Station, United States

*Corresponding author: Prof Carla M. Prado, University of Alberta, 4–002 Li Ka Shing Centre for Health Research Innovation (Office 2-021E), Edmonton, Alberta, T6G 2E1, Canada, +1-780-492-7934, carla.prado@ualberta.ca

Supplementary Table 1. Composition of multivitamins provided to patients with colorectal cancer in the Protein Recommendation to Increase Muscle (PRIMe) study

	**Centrum Men 50+ Complete Multivitamin**	**Nature's Bounty Multivitamin Adult Gummies**
NPN	80043120	80024313
Dose, capsules/day	1	2
**Medical ingredients per dose:**		
Beta-carotene	1800 mcg (3000 IU)	
Biotin	54 mcg	300 mcg
Folate	300 mcg	400 mcg
Niacinamide	16 mg	10 mg
Pantothenic acid	12.5 mg	5 mg
Vitamin A	225 mcg (750 IU)	750 mcg RAE (2500 IU)
Vitamin B_1	4.2 mg	
Vitamin B_2	4.6 mg	
Vitamin B_6	10 mg	2 mg
Vitamin B_{12}	45 mcg	6 mcg
Vitamin C	180 mg	60 mg
Vitamin D	20 mcg (800 IU)	20 mcg (800 IU)
Vitamin E	22.5 mg (50 IU)	6.8 mg AT (15 IU)
Vitamin K1	25 mcg	
Calcium	250 mg	
Choline		40 mcg
Chromium	100 mcg	
Copper	0.5 mg	
Iodine	150 mcg	
Inositol		60 mcg
Iron	2 mg	
Lutein	600 mcg	
Lycopene	600 mcg	
Magnesium	125 mg	
Manganese	3 mg	
Molybdenum	50 mcg	
Selenium	55 mcg	
Zinc	11 mg	5 mg

IU: international units; mcg: microgram; mg: milligram; NPN: natural product number; RAE: retinol activity equivalents.

Supplementary Table 2. Composition of whey protein powder provided to select patients[1] in the Protein Recommendation to Increase Muscle (PRIMe) study

	**Beneprotein®**		**PC Natural Source Whey Protein Isolate Unflavoured Protein**	
Serving size per	7 g		30 g	
**Nutritional Information**	**Amount**	**% Daily Value**	**Amount**	**% Daily Value**
Calories	25		110	
Fat, g	0	0%	0.2	1%
Saturated fat, g			0	0%
Trans Fat, g			0	0%
Cholesterol, mg			0	
Sodium, mg	15	1%	50	2%
Potassium, mg	35	2%	0	0%
Carbohydrate, g	0	0%	2	1%
Sugars, g			1	
Dietary Fibre, g			1	4%
Protein, g	6		25	
Calcium		2%		10%
Iron				2%

[1]n=23 received Beneprotein®; n=6 received PC Natural Source Whey Protein Isolate Unflavoured Protein Drink Mix. g: gram; mg: milligram.

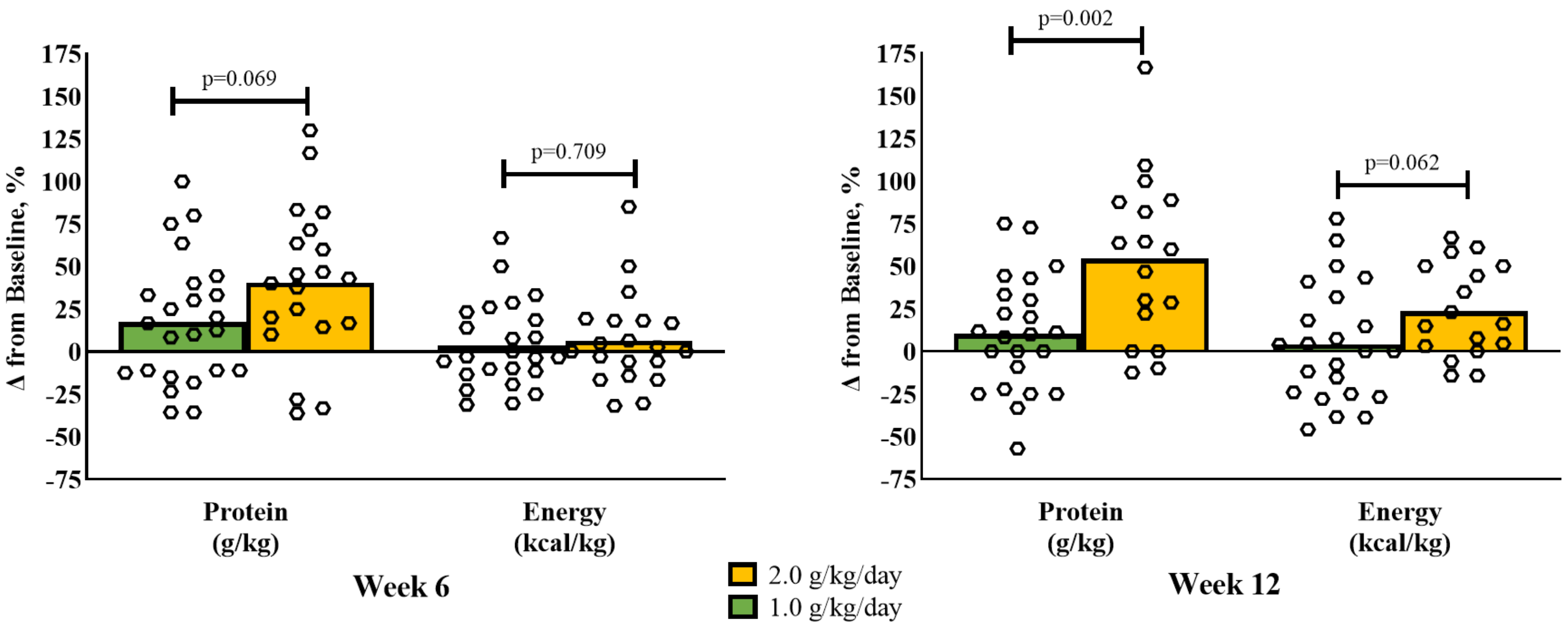

Supplemental Figure 1. Percent change for protein and energy intakes adjusted for body weight from baseline at six and twelve weeks. Boxes represent the mean; dots represent individual patients. Independent samples t-test was used to compare groups for each variable at each time point. All patients who completed the illustrated assessments are included. Six weeks: n=44 (1.0 g/kg/day group: n=24; 2.0 g/kg/day group: n=20); Twelve weeks: n=40 (1.0 g/kg/day group: n=23; 2.0 g/kg/day group: n=17). Δ: change; g/kg: grams per kilogram [body weight]; kcal/kg: kilocalories per kilogram [body weight].

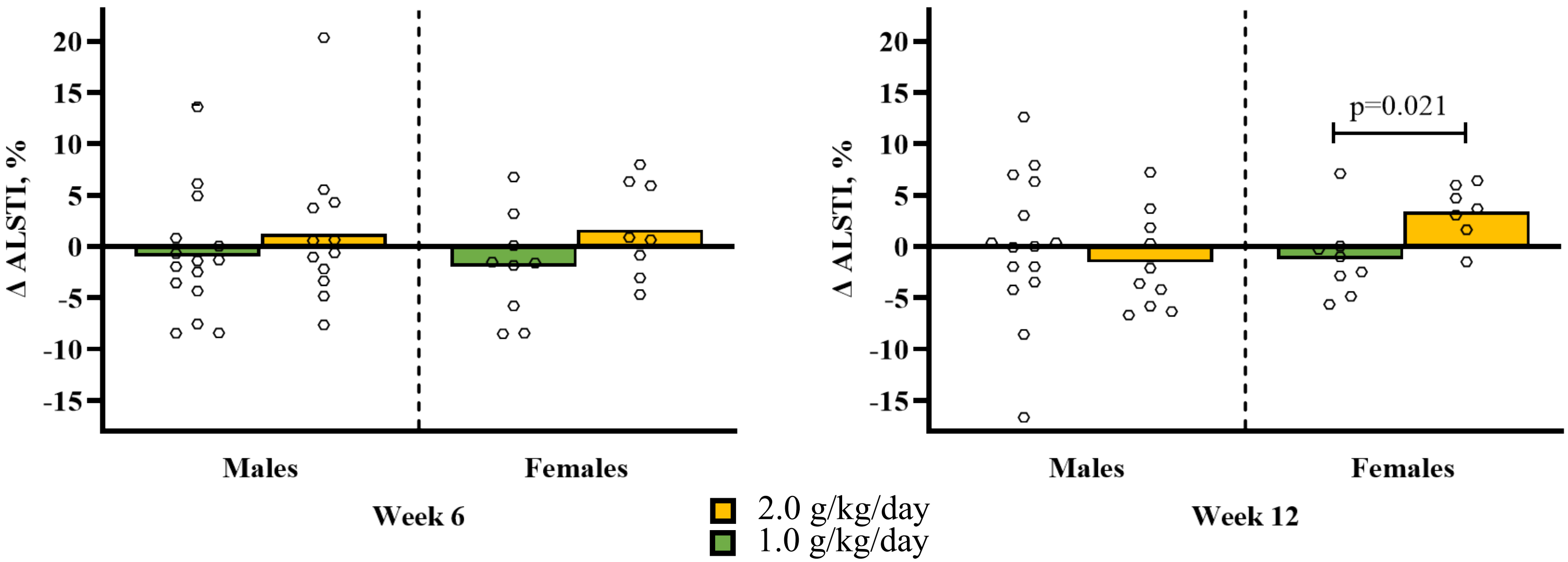

Supplemental Figure 2. Percent change in appendicular lean soft tissue index by study arm and sex from baseline to six and twelve weeks. Each data point represents a patient. Data point with through line represents extreme outlier (significance was not impacted by extreme outlier). Bars represent the group mean. ALSTI: appendicular lean soft tissue index. Six weeks: n=44 (1.0 g/kg/day group: n=24; 2.0 g/kg/day group: n=20); Twelve weeks: n=40 (1.0 g/kg/day group: n=23; 2.0 g/kg/day group: n=17).

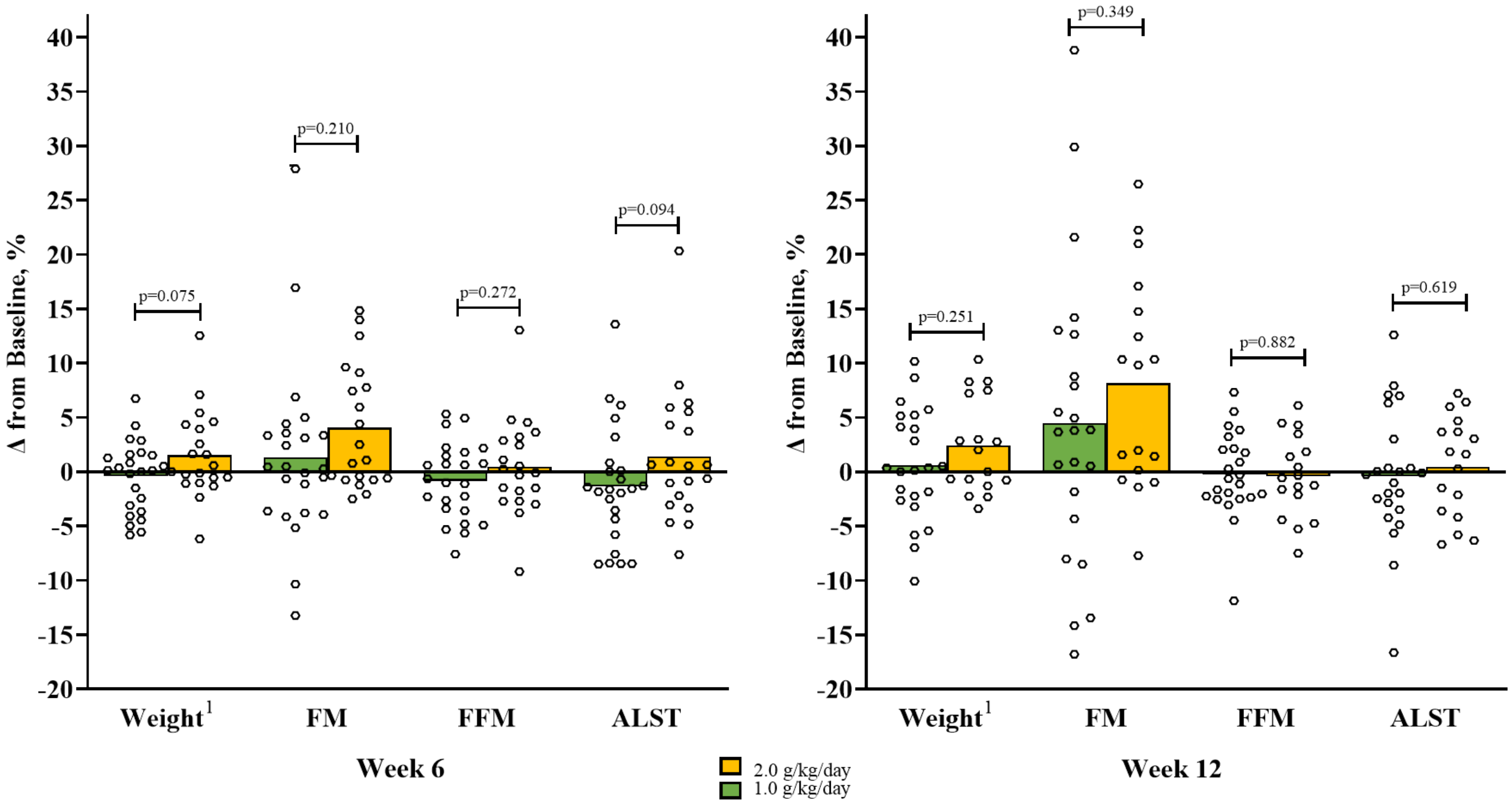

Supplemental Figure 3. Percent change since baseline in weight, fat mass, fat-free mass, and appendicular lean soft tissue at six and twelve weeks. Boxes represent the mean. Data point with through line represents extreme outlier (significance was not impacted by extreme outlier). Independent samples t-test was used to compare groups for each variable at each time point. All patients who completed the illustrated assessments are included. Six weeks: n=44 (1.0 g/kg/day group: n=24; 2.0 g/kg/day group: n=20); Twelve

weeks: n=40 (1.0 g/kg/day group: n=23; 2.0 g/kg/day group: n=17). 1Weight was obtained from dual-energy X-ray absorptiometry scale. Δ: change; ALST: appendicular lean soft tissue; FFM: fat-free mass; FM: fat mass.

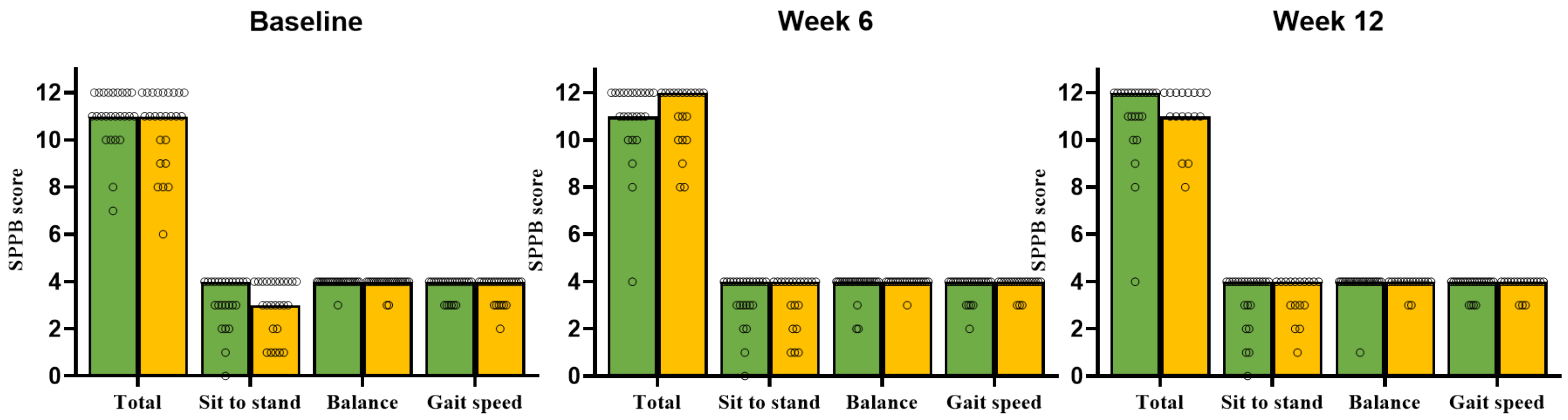

Supplemental Figure 4. Short physical performance battery test scores at baseline, week 6, and week 12. Boxes represent the median; dots represent individual patients. All patients who completed the illustrated assessments are included. Baseline: n=50 (1.0 g/kg/day group: n=25; 2.0 g/kg/day group: n=25); Six weeks: n=44 (1.0 g/kg/day group: n=24; 2.0 g/kg/day group: n=20); Twelve weeks: n=40 (1.0 g/kg/day group: n=23; 2.0 g/kg/day group: n=17). SPPB: short physical performance battery.
